# Streamlined Radiosynthesis of [^18^F]Fluproxadine (AF78): An Unprotected Guanidine Precursor Enables Efficient One-Step, Automation-Ready Labeling for Clinical Use

**DOI:** 10.3390/pharmaceutics18010123

**Published:** 2026-01-19

**Authors:** Xinyu Chen, Kaito Ohta, Hiroyuki Kimura, Yusuke Yagi, Takanori Sasaki, Naoko Nose, Masaru Akehi, Tomohiko Yamane, Rudolf A. Werner, Takahiro Higuchi

**Affiliations:** 1 Nuclear Medicine, Faculty of Medicine, University of Augsburg, 86159 Augsburg, Germany; 2 Department of Nuclear Medicine and Comprehensive Heart Failure Center (DZHI), University Hospital Würzburg, Oberdürrbacher Str. 6, 97080 Würzburg, Germany; 3 Faculty of Medicine, Dentistry and Pharmaceutical Sciences, Okayama University, Okayama 700-8530, Japan; 4 Agency for Health, Safety and Environment, Kyoto University, Kyoto 606-8501, Japan; 5 Department of Radiological Technology, Faculty of Medicinal Science, Kyoto University of Medical Science, Kyoto 606-8501, Japan; 6 Department of Molecular Imaging Research, Kobe City Medical Center General Hospital, Kobe 650-0047, Japan; 7 Department of Nuclear Medicine, LMU Hospital, and German Cancer Consortium (DKTK), Partner Site Munich, Ludwig-Maximilians-University of Munich, 80539 Munich, Germany; 8 Division of Nuclear Medicine and Molecular Imaging, The Russell H Morgan Department of Radiology and Radiological Science, Johns Hopkins University School of Medicine, Baltimore, MD 21205, USA

**Keywords:** norepinephrine transporter, positron emission tomography, [^18^F]AF78, [^18^F]fluproxadine, radiolabeling

## Abstract

**Background/Objectives:** [^18^F]Fluproxadine (formerly [^18^F]AF78) is a PET radiotracer targeting the norepinephrine transporter (NET) with potential applications in cardiac, neurological, and oncological imaging. Its guanidine moiety, while essential for NET binding, presents major radiosynthetic challenges due to high basicity and the harsh deprotection conditions required for protected precursors. Previous methods relied on multistep procedures, strong acids, and complex purification, limiting clinical translation. This study aimed to develop a practical one-step radiosynthesis suitable for routine and automated production. **Methods:** A direct SN2-type nucleophilic [^18^F]fluorination was performed using an unprotected guanidine precursor to eliminate deprotection steps. Reaction parameters, including the base system, solvent composition, precursor concentration, and temperature, were optimized under conventional and microwave heating. Radiochemical conversion (RCC) and operational robustness were evaluated, and purification strategies were assessed for automation compatibility. **Results:** Direct [^18^F]fluorination using the unprotected precursor reduced the total synthesis time to 60–70 min. Optimal conditions employed a *tert*-butanol/acetonitrile (4:1) solvent system with K_2_CO_3_/Kryptofix222, affording RCC up to 33% under conventional heating. Microwave irradiation further improved efficiency, achieving RCC of up to 64% within 1.5 min at 140 °C. The method showed broad tolerance to variations in the base molar ratio and precursor concentration and enabled isocratic HPLC purification. **Conclusions:** This one-step radiosynthesis overcomes longstanding challenges in [^18^F]fluproxadine production by eliminating harsh deprotection and enabling high-yield, automation-ready synthesis, thereby improving clinical feasibility.

## 1. Introduction

**Figure 1:**
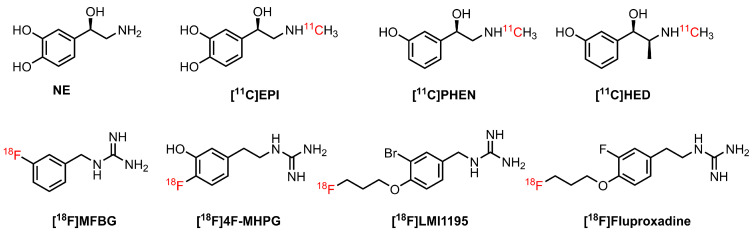
Chemical structures of selected norepinephrine transporter-targeting radiotracers using positron emission tomography.

Previously reported radiotracer [^18^F]fluproxadine (formerly [^18^F]AF78) targeting the norepinephrine transporter (NET) has been investigated in cell studies, rodents, and non-human primates and has proven feasible for selective and specific NET imaging using positron emission tomography (PET) technology (Figure 1) [1,2]. The guanidine moiety provides the strong basicity (pKa 13.6) required for stable accumulation within the storage vesicles of presynaptic sympathetic nerve terminals. At physiological pH, the resulting positive charge facilitates a strong electrostatic interaction with negatively charged phospholipids on the inner surface of the vesicular membranes [3]. In addition, the guanidine moiety is also resistant against metabolism via monoamine oxidase (MAO)—unlike neurotransmitter norepinephrine or previously reported ^11^C-labeled monoamine-based PET tracers such as [^11^C]epinephrine or [^11^C]phenephrine. Although the α-methyl group in [^11^C]*meta*-hydroxyephedrine provides resistance to MAO as well, its continuous leaking out and reuptake mechanism allows for the evaluation of only NET function without involving the transportation into presynaptic nerve storage vesicles [4]. Nevertheless, this strong basicity poses challenges for radiolabeling by potentially promoting precursor elimination side reactions during radiofluorination and by forming stable ion pairs with [^18^F]F^−^, consequently reducing the radiochemical yield (RCY).

For the reasons outlined above, a precursor with a fully protected guanidine moiety was selected, as previously reported [1], due to its enhanced stability during synthesis and resistance to the strong basic conditions required for radiofluorination. Radiolabeling was successfully performed using a two-step, one-pot procedure, achieving a maximum RCY of 27.9%. Despite this success, the approach required harsh acidic deprotection conditions (e.g., 4–6 M HCl or stronger acids like HBr or HI at elevated temperatures), which led to several practical and chemical issues. These included acetamide formation via hydrolysis of acetonitrile (radiolabeling solvent), precipitation formation, retention time shifts during HPLC purification, and incomplete removal of acidic reagents. Moreover, the 1,3,5-triazinane protection group on guanidine showed partial resistance to deprotection, complicating purification due to close retention time overlap with the final product [1].

Further efforts to optimize precursor design using alternative protection strategies highlighted additional limitations. Studies on structurally related phenethylguanidine-based tracers demonstrated that even substituents not directly involved in the labeling mechanism could significantly affect radiochemical performance [5,6,7,8]. In our previous work, meta-substituted analogs of [^18^F]fluproxadine exhibited markedly different RCYs despite identical labeling conditions, underscoring the sensitivity of this scaffold to steric and electronic effects [6]. Attempts to improve stability using tetra-Boc-protected guanidine precursors—successful in the synthesis of [^18^F]MHPG [7,8]—proved unsuitable for the fluproxadine framework, as the bulky protecting groups led to poor stability, low yields, and degradation during synthesis and purification. In contrast to related tracers such as [^18^F]MHPG and [^18^F]MFBG, which tolerate extreme acidic deprotection conditions [8,9,10], the fluproxadine scaffold was prone to decomposition or incomplete deprotection under comparable conditions, further complicating translation to automated synthesis.

These cumulative challenges motivated a fundamental redesign of the precursor strategy. To develop a more robust and automation-compatible radiolabeling process, we transitioned from a protected to an unprotected guanidine precursor. This approach eliminated the need for harsh acidic deprotection, simplified purification, reduced synthesis time, and improved overall reliability. In the present study, we report the development of this streamlined labeling method and the systematic optimization of key parameters—including base, temperature, solvent, precursor concentration, and reagent ratios—to achieve improved radiochemical yield and operational simplicity. In addition, the use of microwave irradiation was explored as a means to further enhance efficiency and reduce reaction time.

## 2. Materials and Methods

### 2.1. General

All reagents were commercially available compounds and were used without further purification unless noted otherwise. ^1^H- and ^13^C-NMR spectra were recorded on a Bruker 400 MHz spectrometer (Billerica, MA, USA). The purity of final products was assessed to be ≥95% by HPLC using a ThermoFisher Vanquish Core system (Waltham, MA, USA) equipped with either a COSMOSIL C18-AR-II (5 µm, 4.6 × 150 mm) or a C18-PAQ (5 µm, 4.6 × 250 mm). The mobile phase consisted of (A) water with 0.1% trifluoroacetic acid (TFA) and (B) acetonitrile with 0.1% TFA, eluted at a flow rate of 1.5 mL/min with the following gradient: 10–80% of phase B over 13 min.

### 2.2. Chemistry

**Scheme 1:**
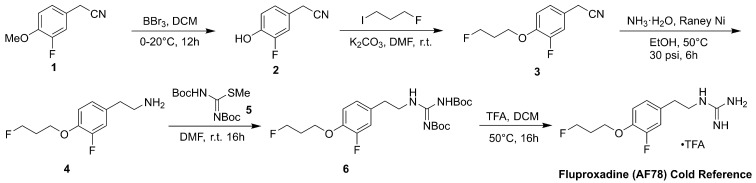
Modified synthetic scheme of cold reference of [^18^F]fluproxadine.

**Scheme 2:**
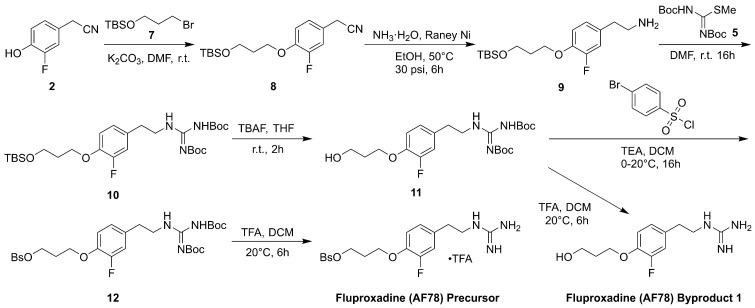
Synthetic scheme of unprotected precursor of [^18^F]fluproxadine and byproduct **1** decomposed during radiolabeling from its precursor.

The cold (non-radioactive) reference standard of [^18^F]fluproxadine was prepared with significant modifications from our previously published procedure [1], reducing the synthetic route from eight to five steps (Scheme 1). The phenethylguanidine core was efficiently constructed in two steps starting from benzylcyanide. This involved a Raney Nickel reduction into phenethylamine, followed by guanidine formation via a nucleophilic reaction with *di-*Boc*-S-*methyl thiourea. The cold reference was obtained after the subsequent deprotection of Boc groups. The precursor (compound used for radiolabeling) with the unprotected guanidine moiety was synthesized with an initial introduction of a *tert*-butyldimethylsilyl (TBS)-protected “tail” structure, which will be converted to a good leaving group for radiofluorination. The phenethylguanidine core structure was established via a similar strategy to the cold reference. Following TBS deprotection, a *para*-bromobenzenesulfonate (Bs) ester was formed to serve as the leaving group for the SN2 radiofluorination step in the radiolabeling process. The unprotected precursor was obtained after the deprotection of Boc groups using mild conditions with TFA in dichloromethane, which did not influence the reactive Bs ester (Scheme 2). In addition, the hydrolysis byproduct of [^18^F]fluproxadine during the radiolabeling process was prepared from an alcohol intermediate, which can be used as a reference for developing a method of HPLC purification. A detailed experimental section and target compound characterization are provided in the Supplementary Materials.

### 2.3. Radiochemistry

#### 2.3.1. General Methods

[^18^F]F^−^ was trapped on a preconditioned Sep-Pak QMA cartridge followed by washing with distilled water (3 mL) to remove [^18^O]water. [^18^F]F^−^ was then eluted from the cartridge with a basic solution into a 1 mL conical vial. The solvent was removed at 110 °C under vacuum and nitrogen flow. Acetonitrile (2 × 0.5 mL) was added to the vial, and the solution was dried azeotropically. Labeling was carried out by adding the solution of the precursor in the selected dry solvent, followed by heating and stirring at 90–140 °C for 10–20 min. The reaction mixture was cooled with water and diluted with 1.5 mL mobile phase for HPLC purification. The fraction at the same retention time of [^18^F]fluproxadine cold standard was collected and radioactivity was measured. An aliquot was removed for quality control on an analytical column for characterization, purity, and molar activity. All the radiochemical conversions (RCCs) presented in the current study are calculated from the radioactivity of collected fractions after HPLC purification with decay correction. For purification, HPLC columns, including COSMOSIL C18-AR-II (5 µm, 6 × 150 mm, Kyoto, Japan), Phenomenex Synergi Hydro-RP (10 µm, 10 × 250 mm, Torrance, CA, USA), and Agilent Zorbax Eclipse XDB-C18 (5 µm, 9.4 × 250 mm, Santa Clara, CA, USA), were used to screen the proper methods of purification. Analytical column COSMOSIL C18-AR-II (5 µm, 4.6 × 150 mm) was used for quality control and molar activity calculation. Different mobile phases, such as water/acetonitrile with TFA, buffer solutions like (NH_4_)_2_CO_3,_ or ion-pair solutions with sodium octanesulfonate, were used for either purification or quality analysis.

#### 2.3.2. Radiolabeling of [^18^F]Fluproxadine (Af78) Using Unprotected Precursor

The [^18^F]fluoride solution (typically started from approximately 2 GBq) was passed through a Waters Sep-Pak Accell Plus QMA Plus Light Cartridge (Waters GmbH, Eschborn, Germany; preconditioned with 5 mL of 0.5 M NaHCO_3_ and 10 mL of Millipore water) before rinsing with 2 mL of pure water. The [^18^F]fluoride was eluted into a 1 mL conical vial by a 600 µL solution of 0.01 M potassium carbonate (6.0 µmol) and Kryptofix_222_ (K_222_, 12 µmol) in 90% methanol/water. The mixture was azeotropically dried at 110 °C for 10 min, followed by a 6 min drying post addition of anhydrous acetonitrile (600 µL). A solution of the precursor (2.4 mg, 4.0 µmol) in a 300 µL anhydrous mixture of *tert*-butanol: acetonitrile (4:1) was added to the vial, and the resulting solution was heated at 110 °C for 15 min. The reaction mixture was let to cool and diluted with 1.5 mL of 30% MeCN/H_2_O (0.1% TFA) and was purified via an HPLC system equipped with a Phenomenex Synergi Hydro-RP column (5 µm, 10ID × 250 mm). Mobile phase: A: water with 0.1% trifluoroacetic acid; B: acetonitrile with 0.1% trifluoroacetic acid; flow rate of 3.0 mL/min; a isocratic 35% of phase B. The fraction containing [^18^F]fluproxadine (10.9 min) was collected, diluted with 20 mL water, and trapped on a Sep-Pak C18 Plus Light cartridge (preconditioned with 5 mL of ethanol and 10 mL of water). The cartridge was washed with 5 mL of water. The product was eluted with 1 mL of ethanol and diluted with saline for further studies. The radiochemical identity, purity, and molar activity of the target radiotracer were assessed using a COSMOSIL 5C_18_-ARII column (5 µm, 4.6 ID × 150 mm) with mobile phases: A: 5 mM sodium octanesulfonate with 1 M H_3_PO_4_ adjusted pH to 3.5; B: acetonitrile; flow rate of 1.5 mL/min and a gradient of 10–60% mobile phase B in 12 min (retention time, 10.6 min).

#### 2.3.3. Microwave-Assisted Radiosynthesis

Following the conventional heating procedures described above, microwave-assisted reactions were performed using a Monowave 400 reactor (Anton Paar GmbH, Ostfildern, Germany). [^18^F]F^−^ trapped on a QMA cartridge was eluted into a G4 reaction vial (minimum volume of 0.5 mL, sealed with silicon septum), containing a 5 mm magnetic stir bar. After azeotropic drying at 110 °C under vacuum and nitrogen flow, the precursor (0.5–2.4 mg in 500 µL of selected solvent) was added to the vial via syringe. The vial was then transferred to the microwave cavity, and the mixture was stirred at 600 rpm while being heated rapidly to the target temperature (110–160 °C). This temperature was maintained for 1.5 min, followed by active cooling to 70 °C using compressed air. The resulting mixture was diluted with the HPLC mobile phase and transferred for purification.

## 3. Results

### 3.1. Preliminary Radiolabeling Condition Screening with Single Solvents

To establish an effective radiolabeling protocol, several key factors were systematically investigated. These included the following: (1) the type of base used for eluting [^18^F]F^−^ from the QMA cartridge, (2) the choice of solvent for both dissolving the precursor and performing the radiofluorination, (3) the concentrations of base and precursor, (4) the molar ratio of base to precursor, and (5) the reaction temperature and duration.

Among these variables, the base and reaction solvent were prioritized for initial optimization. For this initial screening, QMA cartridges were used in their commercially supplied form without further preconditioning to establish a baseline for the elution efficiency of different bases. Common radiolabeling conditions were tested using these cartridges for [^18^F]F^−^ trapping, followed by elution with bases such as Bu_4_NHCO_3_, Et_4_NHCO_3,_ and K_2_CO_3_/K_222_. For each base condition, typical polar aprotic solvents—MeCN and DMSO—were employed to dissolve the precursor (starting with approximately 1 mg in 300 µL) and to conduct radiofluorination. The reaction temperature and duration were fixed at 110 °C for 15 min. All RCC values were calculated from the HPLC-purified fraction and decay-corrected (d.c.).

Under comparable conditions using Bu_4_NHCO_3_ in MeCN, the use of supplied form QMA cartridges resulted in low RCC regardless of the base-to-precursor molar ratio. Similarly, using the stronger base K_2_CO_3_ with K_222_ failed to improve the yield. These low initial values could be attributed to the presence of counter-ions, such as chloride, in the supplied form QMA, which can compete with fluoride and cause a low yield of radiofluorination compared to bicarbonate or carbonate forms after preconditioning. A notable improvement was only observed when the base was switched to Et_4_NHCO_3_ and the precursor concentration was increased; under these conditions, the RCC reached 13%. Moreover, replacing MeCN with DMSO as the reaction solvent did not lead to any further enhancement in RCC (Table 1).

### 3.2. Weaker Bases for Cartridge Elution and Radiofluorination

Managing the balance between nucleophilic substitution and base-mediated side reactions, such as elimination or precursor degradation, is a well-documented challenge in radiofluorination. Initially, we were concerned about the stability of the precursor under basic radiofluorination conditions, particularly due to the high reactivity of the *para*-bromobenzenesulfonate ester moiety. High basicity can facilitate competing elimination processes or degrade sensitive functional groups, thereby reducing the available precursor and lowering the obtainable RCC. Given this sensitivity, we explored strategies to reduce the “harshness” of the reaction environment, consistent with “low-base” or “neutralized-base” concepts reported in the literature [11,12].

Several attempts were made using weaker bases, such as incorporating dihydrophosphate in the elution mixture along with carbonate or bicarbonate [13]. However, these conditions either resulted in poor elution efficiency of [^18^F]F^−^ from the QMA cartridge—due to insufficient basicity—or led to low RCCs. To overcome these limitations while still minimizing basicity, we adopted a modified approach: [^18^F]F^−^ was eluted using K_2_CO_3_ to ensure efficient recovery, but oxalic acid was preloaded into the reaction vial to partially neutralize the carbonate, thereby reducing the effective basicity without introducing competing nucleophiles [14]. Despite this modification, the resulting RCCs remained suboptimal. This was likely due to the poor solubility of the resulting mixed potassium salts in MeCN. Subsequent attempts to enhance solubility by adding the bulky protic solvent tBuOH—a technique known to stabilize anions and temper basicity—did not yield significant improvements at this stage (Table 2).

### 3.3. Mixed Solvents Were Chosen for Radiofluorination

Building upon the previous findings, we next turned our attention to the potential benefits of mixed solvent systems—particularly those incorporating protic solvents—to further enhance RCC. This direction was motivated both by prior reports on the successful use of alcohol-containing solvent mixtures for the radiolabeling of LMI1195 [15] and by mechanistic studies highlighting the role of protic solvents in promoting SN2 reactions [11].

To investigate this, we evaluated a range of alcohol-based protic solvents—specifically nBuOH, iBuOH, and tBuOH—in combination with MeCN, with a particular focus on 4:1 (*v*/*v*) solvent ratios. Among the various combinations tested, the mixture of tBuOH/MeCN showed the most promising performance in this initial experiment, delivering an RCY of 33% using K_2_CO_3_ as the base; however, as some of the results are based on a single experiment, it should be considered preliminary. Notably, the structurally similar mixture iBuOH/MeCN (4:1) gave a dramatically lower RCC of only 0.5%, highlighting the sensitivity of the system to subtle changes in solvent properties.

Following the promising results of the tBuOH/MeCN system, we systematically evaluated the impact of various bases under these optimized solvent conditions. A range of bases with varying strengths was examined, including K_2_CO_3_, Et_4_NHCO_3_, Bu_4_NHCO_3_, and Bu_4_NH_2_PO_4_. To maintain sufficient nucleophilicity across different basicity levels, the base-to-precursor ratio was adjusted: 1.5 equivalents for K_2_CO_3_, 5 equivalents for the bicarbonates, and 10 equivalents for Bu_4_NH_2_PO_4_. The strongest base, K_2_CO_3_, yielded the highest RCC, while the bicarbonate salts provided moderate yields (~20%). Conversely, Bu_4_NH_2_PO_4_ resulted in a significantly lower RCC (4%), which may be attributed to its limited solubility in the predominantly organic tBuOH/MeCN mixture. These results indicate that both basic strength and solubility in mixed protic–aprotic media are decisive factors for efficient radiofluorination (Table 3).

### 3.4. Systematic Investigation of Reaction Conditions

**Figure 2:**
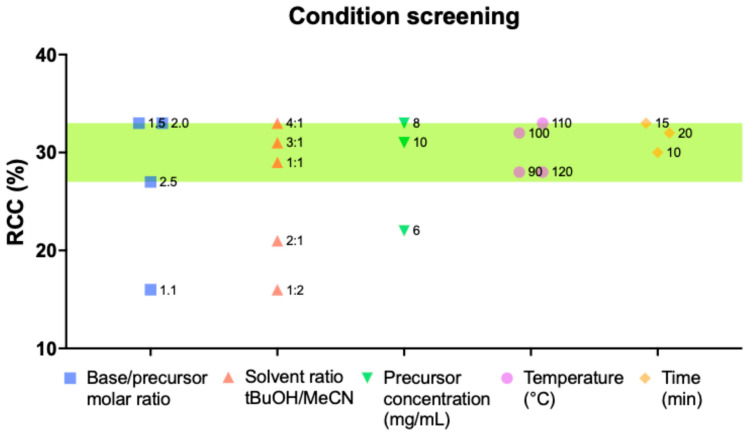
Summary of systematic investigation of labeling conditions. The green band highlights the range of conditions for optimal radiochemical conversion (RCC) (30 ± 3%). Detailed conditions are listed as tables and provided in the Supplementary Materials.

A comprehensive optimization of the radiofluorination conditions was performed starting from baseline conditions using 1.5 molar equivalents of K_2_CO_3_/K_222_ as the base/eluent system together with a concentration of 2.4 mg of precursor in tBuOH/MeCN 4:1 (*v*/*v*) at 110 °C for 15 min, with the goal of maximizing RCC (Figure 2, Table S1). First, among the base-to-precursor ratios, 1.5 to 2 equivalents of K_2_CO_3_ provided the highest RCC of 33%. Lowering the base to 1.1 equivalents resulted in a reduced RCC of 16%, while increasing it beyond 2 equivalents resulted in the RCC starting to decrease, suggesting a negative impact on the efficiency of incorporation or decomposition of product with an excessive base.

Second, the solvent ratio also had a notable influence on labeling efficiency. While the 4:1 tBuOH/MeCN mixture gave optimal results, decreasing the proportion to 3:1 still maintained the RCC (31%). However, further reduction in the tBuOH content led to significant drops in RCC—down to 21% with a 2:1 ratio and as low as 16% with a 1:2 ratio—indicating that a higher proportion of protic solvent is favorable.

Third, the concentration of precursor was investigated to facilitate its transfer to automatic radiosynthesis using a module. This study aimed to identify the lowest concentration allowable while preserving the RCC under current labeling conditions, with the base-to-precursor ratio kept constant throughout. The results revealed that even with a slight reduction in precursor concentration, the RCC dropped from 33% to 22%. In contrast, increasing the precursor concentration while maintaining the same ratio preserved the RCC, as expected.

Last, reaction temperature and time variations showed that 110 °C for 15 min was optimal, though similar RCCs were obtained with slight adjustments in temperature (90–120 °C) or reaction time (10–20 min), indicating some tolerance in these parameters.

### 3.5. Exploration of Radiolabeling Using Microwave-Assisted Reaction

The use of microwave-assisted heating enabled rapid attainment of high temperatures and uniform energy distribution, facilitating efficient labeling within a short reaction time. Under the established labeling conditions using K_2_CO_3_ (1.5 eq.) in a 4:1 tBuOH/MeCN ratio, a brief heating period of 1.5 min at 110 °C already achieved 21% RCC, compared to 33% under conventional heating. Upon increasing the temperature to 140 °C, a 60% RCC was achieved in only 1.5 min with high reproducibility. In contrast, conventional heating using an aluminum block required 20 min to reach roughly half that yield (33%). Further increasing the temperature to 160 °C led to a decline in RCC (40%), likely due to thermal decomposition of the product; however, this remained superior to the results obtained with conventional heating methods.

As a comparison, tBuOH (b.p. 82 °C) was replaced with higher boiling point protic solvents to evaluate the effect of temperature and steric bulk. While iBuOH (b.p. 108 °C) did not improve the RCC, the more sterically hindered thexyl alcohol (b.p. 118 °C) achieved a 43% RCC under identical conditions (Table 4). This indicates that while higher temperatures are beneficial, the preservation of the SN2 pathway requires high steric bulk in the protic components to suppress side reactions. These results suggest that the increased RCCs are not solely a function of temperature, but rather a synergistic effect of microwave-induced acceleration and optimized solvent composition.

### 3.6. Further Optimization at 140 °C but with Lower Precursor Concentration

Lastly, we further explored the potential for translating the protocol to an automatic synthetic module with an even higher temperature and a lower concentration of precursor. Although these concepts are against the conclusion drawn from early experiments, the results from microwave-assisted radiolabeling provided some hints at the possibility of such optimization. Since the reaction will take a longer time than using a microwave reactor, the reaction time was set to 10 min, still at 140 °C. Several conditions have been altered again to check the impact, mainly focusing on the base-sensitive conditions using various QMA preconditioning and elution strategies, along with different solvents (Table 5). At 140 °C, similar to 120 °C, it still delivered a moderate RCC of 27% but with only 1.0 instead of an 8 mg/mL concentration of precursor. Compared to Table 1, a lower yield resulted from a lower concentration of precursor, which can be compromised by increasing the reaction temperature. Alternative base systems using organic quaternary ammonium salts in DMSO resulted in low RCCs. Replacing tBuOH with thexyl alcohol due to its high boiling point yielded up to 38% RCC—one of the most efficient setups under conventional heating with much less precursor.

### 3.7. Optimization of Hplc Method for Product Purification and Quality Analysis

**Figure 3:**
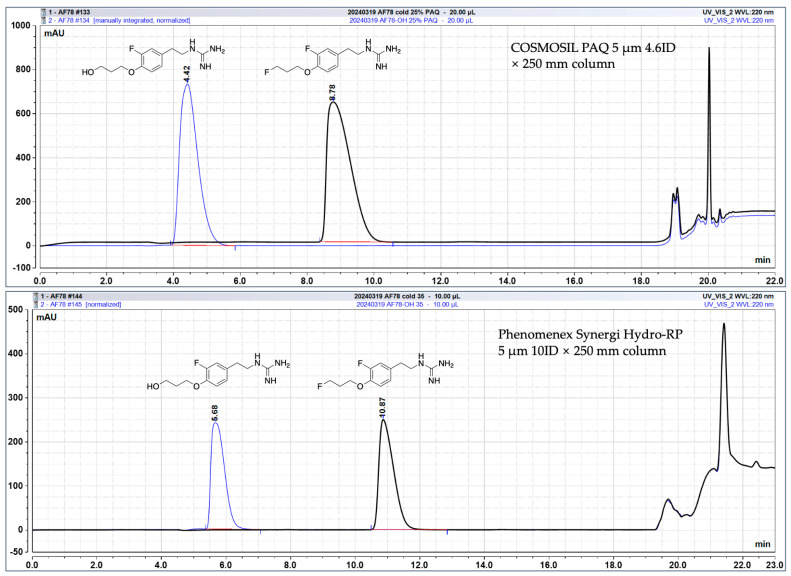
Representative HPLC chromatogram for purification of [^18^F]fluproxadine (black) from byproduct **1** (blue) decomposed during radiolabeling from its precursor with either COSMOSIL PAQ 5 µm 4.6ID × 250 mm column (**top**, 25% isocratic MeCN/H_2_O with 0.1%TFA, 3 mL/min flow, [^18^F]fluproxadine RT 8.78 min) or Phenomenex Synergi Hydro-RP 5 µm 10ID × 250 mm column (**bottom**, 35% isocratic MeCN/H_2_O with 0.1%TFA, 3 mL/min flow, [^18^F]fluproxadine RT 10.87 min).

**Figure 4:**
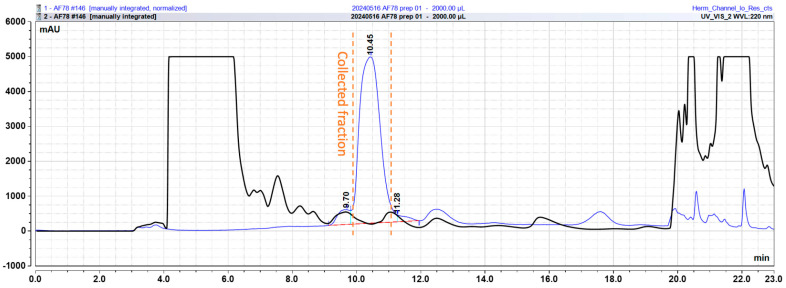
Representative HPLC chromatogram for purification of [^18^F]fluproxadine after radiolabeling, showing the overlapping UV (220 nm, black) and radioactive detector (blue) channels. HPLC condition: COSMOSIL PAQ 5 µm 4.6ID × 250 mm column, 35% isocratic MeCN/H_2_O with 0.1%TFA, 3 mL/min flow.

The original HPLC method to purify [^18^F]fluproxadine after radiofluorination and deprotection under acidic conditions was a gradient method with 10–80% of phase B (MeCN with 0.1% TFA) in 13 min, because the partially deprotected intermediate was very close to the product [1]. An isocratic method using 25–35% of MeCN/water (0.1% TFA) allows the separation of [^18^F]fluproxadine from the unprotected precursor as well as the potential decomposed byproduct **1** due to hydrolysis of the precursor. Both COSMOSIL PAQ and Phenomenex Synergi Hydro-RP columns have been tried. Both showed good separation of these two peaks (Figure 3). Furthermore, two byproducts also possibly formed during the basic radiolabeling conditions, namely ethylamine or urea, were also prepared [16]. Both showed retention times on the Synergi Hydro-RP column well separated from the peak of [^18^F]fluproxadine (Figure S1). An exemplary HPLC graph for purification of [^18^F]fluproxadine after a typical radiolabeling procedure as described above is illustrated in Figure 4.

**Figure 5:**
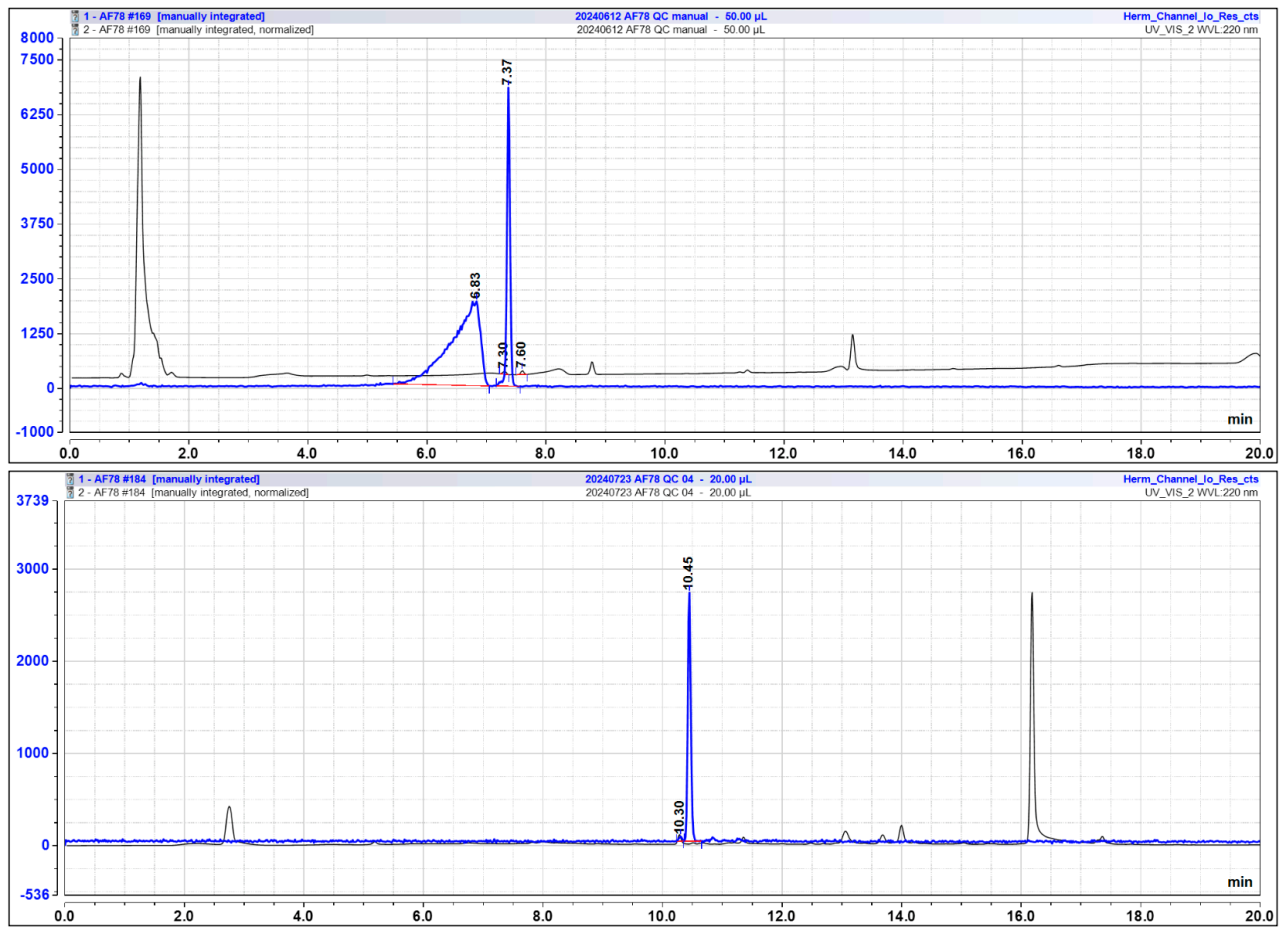
Representative analytical HPLC chromatograms of [^18^F]fluproxadine during quality control before (**top**) and after (**bottom**) optimization with an ion-pair reagent, showing overlapping UV (220 nm, black) and radioactive detector (blue) channels. **Top** HPLC condition: COSMOSIL ARII 4.6 × 150 mm, 1.5 mL/min flow, A: H_2_O (0.1% TFA), B: MeCN (0.1% TFA), 10–80% B in 13 min. **Bottom** HPLC condition: COSMOSIL ARII 4.6 × 150 mm, 1.5 mL/min flow, A: 5 mM sodium octanesulfonate with 1M H_3_PO_4_ adjusted pH to 3.5, B: MeCN, 10–80% B in 13 min.

In addition to the conventional HPLC method for the quality control of [^18^F]fluproxadine, an analytical method using an ion-pair reagent has been developed for accurate quantitative analysis of the product (Figure 5). As shown in the top panel of Figure 5, injection of a higher quantity of [^18^F]fluproxadine led to an apparent splitting of the product peak, with a broad peak (RT 6.83 min) eluting earlier than the peak (RT 7.37 min) corresponding to the cold reference standard. Although this feature was initially suspected to arise from [^18^F]F^−^, the preceding thin-layer chromatography results confirmed that no free fluoride was present, and the peak was therefore attributed to the tracer itself. Upon addition of the ion-pair reagent sodium octanesulfonate, the product peak remained sharp and symmetric even at higher injected activities, enabling more reliable quantitative analysis.

## 4. Discussion

**Scheme 3:**
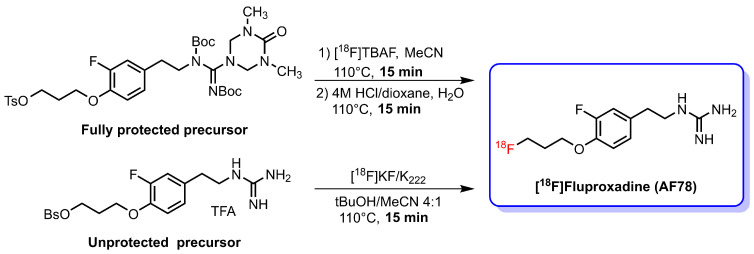
Comparison of [^18^F]fluproxadine radiosynthesis: Previously fully protected vs. new unprotected precursor under typical radiolabeling conditions.

The optimization of the radiolabeling process for [^18^F]fluproxadine using an unprotected precursor has yielded significant advancements over previously reported methods employing fully protected guanidine structures (Scheme 3, Figure S3). The development of a streamlined and robust one-step radiolabeling procedure presents multiple advantages, including shorter synthesis time, higher RCC, simplified purification, and improved compatibility with automated synthesis modules.

### 4.1. Advantages of the Unprotected Precursor Approach

The transition to an unprotected guanidine precursor represents a key practical advancement of this work. The selection of the bromo-substituted derivative as the leaving group was central to this strategy, providing an optimal balance between stability and reactivity. While triflate or nitrobenzenesulfonyl analogs are more commonly used due to their high leaving group ability, they often exhibit limited shelf-life and high sensitivity to moisture or thermal degradation. In contrast, the bromo-substituted precursor demonstrated excellent long-term stability under ambient storage conditions while maintaining sufficient electron-withdrawing strength to facilitate efficient nucleophilic substitution.

Beyond improving radiochemical yield, this strategy significantly simplifies the radiosynthesis by eliminating harsh acidic deprotection and neutralization steps, thereby minimizing product degradation and HPLC variability. This streamlined workflow enhances operational robustness and reduces synthesis time—factors critical for routine clinical production. Furthermore, the reduction in handling steps is particularly advantageous for automated platforms, as it improves reproducibility and prevents equipment corrosion. Ultimately, abandoning conventional protection–deprotection strategies in favor of an unprotected precursor proves both chemically viable and practically superior for the production of [^18^F]fluproxadine.

### 4.2. Base and Solvent Effects in Sn2 Radiolabeling

Among all the factors that have been investigated in the current study, the base used to elute [^18^F]F^−^ from the QMA cartridge and the solvent used for precursor dissolution and radiofluorination demonstrated a significant impact on RCY.

We have explored the impact of base selection from the early stages. Unexpectedly, the results revealed that the precursor is relatively stable against strong bases such as K_2_CO_3_, especially when using a mixed solvent system for radiofluorination. Attempts to replicate the success of mixed solvents using common solvents, such as DMSO or MeCN, with weakly basic eluents, e.g., PO_3_^−^/Et_4_HCO_3_, Bu_4_NH_2_PO_4,_ that have been reported to produce high RCC for base-sensitive precursors [17], did not yield comparable results for [^18^F]fluproxadine. Moreover, lower RCC using a weaker base such as Bu_4_NH_2_PO_4_ may also result from its poorer solubility in the solvent used. These findings underscore the importance of system-specific optimization rather than universal adoption of previously successful conditions.

To improve the RCC of the SN2-type radiofluorination, we systematically investigated the influence of solvent composition and base strength. Drawing on previous findings [11,15], we evaluated mixed solvents incorporating protic alcohols (tBuOH, iBuOH, nBuOH) with acetonitrile. Among these, tBuOH showed the most promising results, likely due to its steric properties and ability to modulate solvation and transition stabilization in SN2 reactions, thus enhancing nucleophilic substitution.

Under the strongly basic and elevated-temperature conditions required for nucleophilic radiofluorination, competing pathways—particularly elimination (E1/E2) and hydrolytic decomposition—can significantly affect product distribution. This is especially critical in radiochemical systems, where subtle changes in solvent polarity and proticity can disproportionately influence reaction selectivity.

In predominantly aprotic environments, hydrolytic pathways are minimized; however, the combination of heat and a strong base favors elimination reactions, leading to alkene byproducts. Introducing a protic co-solvent such as tBuOH provides a means to rebalance these competing processes. In a 4:1 (*v*/*v*) tBuOH/MeCN solvent system, the solvent environment is dominated by a polar protic character, which partially solvates anions and moderates effective base strength. This reduces aggressive β-elimination while still maintaining sufficient nucleophilicity of fluoride to promote SN2 substitution.

At the same time, the minor MeCN component plays a crucial enabling role. Acetonitrile improves precursor solubility and facilitates effective complexation of potassium by K222, thereby minimizing ion pairing and enhancing the availability of “free” fluoride. This combination supports efficient backside attack at the primary substrate, favoring SN2 radiofluorination over both hydrolysis and elimination pathways.

Although the protic nature of tBuOH slightly attenuates fluoride basicity, it does not shift the mechanism toward SN1, which remains disfavored due to the instability of a primary carbocation intermediate. Instead, the solvent mixture suppresses ion-pairing-induced aggregation and excessive base reactivity that would otherwise promote E2 elimination. As a result, alkene formation is minimized, and ester hydrolysis is reduced relative to more strongly basic, aprotic systems.

Overall, the 4:1 tBuOH/MeCN solvent system provides a balanced microenvironment that enhances fluoride nucleophilicity while tempering base strength. This dual effect shifts the reaction landscape toward productive SN2 radiofluorination, limits competitive elimination and hydrolytic degradation, and ultimately contributes to improved radiochemical yield and precursor stability.

The use of tBuOH as a co-solvent proved more advantageous than higher boiling alternatives like thexyl alcohol. Although thexyl alcohol yielded similar or slightly improved RCCs—likely due to its higher boiling point and the ability of its bulky alkyl chain to enhance fluoride nucleophilicity through specific solvation [11,18]—it presented significant practical drawbacks. Its low volatility and lipophilicity led to persistent residual solvent issues during drying and quality control, as well as suboptimal HPLC elution profiles. 2-Methyl-2-butanol (b.p. 102 °C) could be a potential alternative, while its lower boiling point relative to thexyl alcohol might facilitate easier removal before HPLC purification. Ultimately, tBuOH remains the preferred co-solvent, offering a superior balance of performance and ease of downstream handling, particularly concerning purification and the avoidance of potential toxicity risks.

### 4.3. Optimal Radiolabeling Working Window (Molar Ratio, Time, Temperature, Concentration)

In contrast to the variation in RCCs at the early stage of condition investigation, while establishing the baseline protocol using K_2_CO_3_/K_222_ in tBuOH/MeCN (4:1) for radiolabeling of the unprotected precursor of [^18^F]fluproxadine, it showed unexpected stability under these basic conditions, contrary to prior concerns regarding base sensitivity. A relatively broad working window could be seen, as highlighted in green in Figure 2. An RCC of 30 ± 3% could be achieved, while changing the radiolabeling conditions within a certain range, including ~1.5–2.5 molar equivalents of base-to-precursor, up to 50% of tBuOH mixture with MeCN, 8–10 mg/mL of precursor, and 90–120 °C of reaction temperature. Optimal yields were consistently achieved using 1.5–2.0 molar equivalents of K_2_CO_3_/K_222_ relative to the precursor. Too little base resulted in incomplete elution of [^18^F]F^−^ from QMA cartridges, while excessive base did not adversely affect yields, highlighting a broad working window. With conventional heating, similar RCY could be obtained at almost all the temperatures tested. A similar pattern also applies to the reaction time, which demonstrated a relatively fast radiolabeling mechanism under these conditions. Only a lower concentration of precursors and too low proportion of tBuOH (<50%) led to a significant decrease in RCC.

### 4.4. Temperature and Heating Method Optimization

In contrast to the relatively broad working window using conventional heating via aluminum blocks, a change in heating methods significantly influenced radiochemical outcomes. Microwave-assisted heating allowed rapid temperature ramp-up and uniform energy distribution, achieving excellent yields within only 1.5 min at 140 °C. Compared to conventional aluminum block heating at 110 °C for 15 min, microwave heating more than doubled the yield, even outperforming 10 min heating at 140 °C via aluminum blocks. These findings align with previous studies, indicating that microwave heating can enhance conversion and reduce byproduct formation [19,20]. Moreover, increasing the temperature but shortening the reaction time (e.g., 140 °C for 10 min vs. lower concentrations) improved RCC even with lower precursor amounts, further demonstrating the efficiency and precursor stability under controlled thermal stress. The significant improvements in RCY achieved here highlight the potential utility of integrated microwave-assisted modules in radiopharmacy. Although the commercial development of such units would require further industry investment, the efficiency gains observed in this work suggest they could become a valuable standard for the rapid synthesis of base-sensitive tracers.

### 4.5. Automation and Molar Activity Considerations

The streamlined protocol developed here is particularly amenable to automated synthesis. The switch from gradient to isocratic HPLC purification, compatibility with isocratic pumps commonly found in synthesis modules, and elimination of volatile corrosive acids all support the robustness of this method for clinical translation.

One challenge that remains is the relatively low molar activity observed in the manual syntheses. In the present study, this is primarily attributed to the comparatively low starting radioactivity (typically ~2 GBq), which is constrained by local laboratory radiation safety regulations. Notably, these low values are largely a mathematical consequence of the low starting activity used during method development. At these activity levels, even small amounts of non-radioactive carrier present in reagents or introduced during handling can have a proportionally greater impact on the final molar activity. Consequently, the current molar activity data should be viewed as a baseline for small-scale manual preparations rather than an upper limit of the methodology. It is expected that higher starting activities in a full-scale automated setting will lead to a significant improvement in molar activity.

From the perspective of automation, several practical considerations merit discussion. While the current purification strategy is compatible with isocratic operation, the use of TFA in the HPLC system may present limitations for routine clinical production, particularly with respect to module compatibility, long-term maintenance, and downstream formulation requirements. Future efforts should therefore focus on developing fully biocompatible isocratic buffer systems that avoid strong acids and are better aligned with clinical radiopharmacy practice. In this context, ethanol-based mobile phases and buffer systems represent especially attractive alternatives, as they combine compatibility with automated modules, regulatory acceptance, and direct suitability for injectable formulations.

## 5. Conclusions

An unprotected precursor of NET-targeting PET radiotracer [^18^F]fluproxadine has been evaluated to replace the original guanidine fully protected precursor. The radiolabeling conditions to prepare [^18^F]fluproxadine have been investigated, including base, temperature, concentration of the reaction, and different solvents. Among these factors, the solvent plays the most important role in the yield, with tBuOH/MeCN (4:1) demonstrating the most tolerable one against changes in other conditions while still retaining RCC in a good range. Radiofluorination using a microwave reactor at a higher temperature greatly improved RCC, although such an application is limited to a manual labeling protocol. The current investigation on the reaction factors paves the way for further transfer to radiolabeling suitable for clinical application using automatic synthesizers.

## Figures and Tables

**Table 1:** Initial radiolabeling condition screening. Unless otherwise specified, all reactions are at 110 °C for 15 min.

Base/Elution from QMA	Solvent	Amount/Concentration of Precursor	RCC (d.c.)
Bu_4_NHCO_3_ (5 eq.)	300 µL MeCN	1 mg (3.3 mg/mL)	None ^#^
Bu_4_NHCO_3_ (3 eq.)	300 µL MeCN	1 mg (3.3 mg/mL)	None ^#^
Bu_4_NHCO_3_ (5 eq.)	300 µL MeCN	0.9 mg (3.0 mg/mL)	3.3%
K_2_CO_3_ (2.5 eq.), K_222_	300 µL MeCN	1.5 mg (5.0 mg/mL)	4.3%
Et_4_NHCO_3_ (4.6 eq.) in MeOH	200 µL MeCN	1.8 mg (9.0 mg/mL)	13.0%
K_2_CO_3_ (1.5 eq.), K_222_	300 µL DMSO	2.4 mg (8.0 mg/mL)	0.6% (*n* = 2)

^#^ Reaction at 90 °C.

**Table 2:** Radiolabeling condition screening with conditions reported for base-sensitive precursor.

QMA Preconditioning	Base/Elution from QMA	Solvent	Amount/Concentration of Precursor	RCC (d.c.)
Without	K_2_CO_3_ (1.75 eq.), KH_2_PO_4_ (1.75 eq.), K_222_^#^	200 µL MeCN	1.6 mg (8.0 mg/mL)	7.9% (*n* = 2)
5 mL 0.5 M NaHCO_3_, 10 mL water	Bu_4_NHCO_3_ (5 eq.), K_2_HPO_4_ (10 eq.)	300 µL MeCN	1 mg (3.3 mg/mL)	None
5 mL 0.5 M NaHCO_3_, 10 mL water	K_2_CO_3_ (2.5 eq.), K_222_ 15 µL 0.5 M oxalic acid preloaded	300 µL 4:1 tBuOH/MeCN	2.4 mg (8.0 mg/mL)	None

^#^ Radioactivity from QMA could not be totally eluted. Majority side products.

**Table 3:** Radiolabeling condition screening using mixed solvents followed by different bases. All the reactions were performed at 110 °C for 15 min using an aluminum block for heating.

QMA Preconditioning	Base/Elution from QMA	Solvent	Amount/Concentration of Precursor	RCC (d.c.)
5 mL 0.5 M NaHCO_3_, 10 mL water	Et_4_NHCO_3_ (4.6 eq.)	200 µL 2:1 **DMA/nBuOH**	1.6 mg (8.0 mg/mL)	6.7%
	K_2_CO_3_ (1.5 eq.), K_222_ (3 eq.)	300 µL 4:1 **iBuOH**/MeCN	2.4 mg (8.0 mg/mL)	0.5%
	K_2_CO_3_ (1.5 eq.), K_222_ (3 eq.)	300 µL 4:1 tBuOH/MeCN	2.4 mg (8.0 mg/mL)	33%
	**Bu_4_NHCO_3_** (5 eq.)	300 µL 4:1 tBuOH/MeCN	2.4 mg (8.0 mg/mL)	21%
	**Et_4_NHCO_3_** (5 eq.)	300 µL 4:1 tBuOH/MeCN	2.4 mg (8.0 mg/mL)	24%
	**Bu_4_NH_2_PO_4_** (10 eq.) ^#^	300 µL 4:1 tBuOH/MeCN	2.4 mg (8.0 mg/mL)	4%

^#^ Additional water (150 µL) was added to dissolve the salt in 600 µL of 90% MeOH as used for other bases.

**Table 4:** Radiolabeling using a microwave reactor. All QMA cartridges were preconditioned using 5 mL of 0.5 mM NaHCO_3_ and 10 mL of H_2_O.

Base/Elution from QMA ^#^	Solvent	Amount/Concentration of Precursor	Temperature/Time	RCC (d.c.)
K_2_CO_3_ (1.5 eq.), K_222_ (3 eq.)	500 µL 4:1 tBuOH/MeCN	2.4 mg (4.8 mg/mL)	**110** °C, 1.5 min	21%
	500 µL 4:1 tBuOH/MeCN	2.4 mg (4.8 mg/mL)	**140** °C, 1.5 min	60% (*n* = 2)
	500 µL 4:1 tBuOH/MeCN	2.4 mg (4.8 mg/mL)	**160** °C, 1.5 min	40%
	500 µL 4:1 **iBuOH**/MeCN	2.4 mg (4.8 mg/mL)	140 °C, 1.5 min	5%
	500 µL 4:1 **thexyl alcohol**/MeCN	2.4 mg (4.8 mg/mL)	140 °C, 1.5 min	43%

^#^ QMA cartridges preconditioned by 5 mL 0.5 M NaHCO_3_ and 10 mL water.

**Table 5:** Radiolabeling results with various conditions but under conventional heating at 140 °C for 10 min.

QMA Preconditioning	Base/Elution from QMA	Solvent	Amount/Concentration of Precursor	RCC (d.c.)
5 mL 0.5 M NaHCO_3_, 10 mL water	K_2_CO_3_ (1.5 eq.), K_222_ (3 eq.)	500 µL 4:1 tBuOH/MeCN	0.5 mg (1.0 mg/mL)	27%
5 mL 0.5 M Na_3_PO_4_, 10 mL water	Et_4_NHCO_3_ (10 eq.)	500 µL DMSO	0.5 mg (1.0 mg/mL)	5.6%
5 mL 0.5 M NaHCO_3_, 10 mL water	Bu_4_NH_2_PO_4_ (10 eq.)	500 µL DMSO	0.5 mg (1.0 mg/mL)	16%
5 mL 0.5 M Na_3_PO_4_, 10 mL water	Bu_4_NOTf (10 eq.)	500 µL 4:1 thexyl alcohol/MeCN	0.5 mg (1.0 mg/mL)	38%
5 mL 0.5 M NaHCO_3_, 10 mL water	Bu_4_NHCO_3_ (10 eq.)	500 µL 4:1 thexyl alcohol/MeCN	0.5 mg (1.0 mg/mL)	31%

## Data Availability

The original contributions presented in this study are included in the article and Supplementary Material. Further inquiries can be directed to the corresponding author.

## Supplementary Materials

The following supporting information can be downloaded at: https://www.mdpi.com/article/10.3390/pharmaceutics18010123/s1.

## Abbreviations

PET	positron emission tomography
NET	norepinepherine transporter
RCY	radiochemical yield
RCC	radiochemical conversion
