# Supplementary material for: Streamlined Radiosynthesis of [18F]Fluproxadine (AF78): An Unprotected Guanidine Precursor Enables Efficient One-Step, Automation-Ready Labeling for Clinical Use
Source: Pharmaceutics. 2026 Jan 19;18(1):123. doi: 10.3390/pharmaceutics18010123 (PMC12844956; doi:10.3390/pharmaceutics18010123)
Supplement: Supplementary file 1 [file pharmaceutics-18-00123-s001.zip › pharmaceutics-4076487-supplementary.pdf]

Supplementary Materials

Streamlined Radiosynthesis of [^{18}F]Fluproxadine (AF78): An Unprotected Guanidine Precursor Enables Efficient One-Step, Automation-Ready Labeling for Clinical Use

Xinyu Chen [1], Kaito Ohta [2,3], Hiroyuki Kimura [4], Yusuke Yagi [3,5], Takanori Sasaki [3], Naoko Nose [3], Masaru Akehi [3], Tomohiko Yamane [6], Rudolf A. Werner [7,8] and Takahiro Higuchi [2,3,*]

1 Nuclear Medicine, Faculty of Medicine, University of Augsburg, 86159 Augsburg, Germany; chen_x@ukw.de
2 Department of Nuclear Medicine and Comprehensive Heart Failure Center (DZHI), University Hospital Würzburg, Oberdürrbacher Str. 6, 97080 Würzburg, Germany; arutema929@gmail.com
3 Faculty of Medicine, Dentistry and Pharmaceutical Sciences, Okayama University, Okayama 700-8530, Japan; yusuke_y@ms.kyoto-phu.ac.jp (Y.Y.); t-sasaki@cc.okayama-u.ac.jp (T.S.); ptot12h6@okayama-u.ac.jp (N.N.); akehi@cc.okayama-u.ac.jp (M.A.)
4 Agency for Health, Safety and Environment, Kyoto University, Kyoto 606-8501, Japan; kimura.hiroyuki.4u@kyoto-u.ac.jp
5 Department of Radiological Technology, Faculty of Medicinal Science, Kyoto University of Medical Science, Kyoto 606-8501, Japan
6 Department of Molecular Imaging Research, Kobe City Medical Center General Hospital, Kobe 650-0047, Japan; tomohiko_yamane@kcho.jp
7 Department of Nuclear Medicine, LMU Hospital, and German Cancer Consortium (DKTK), Partner Site Munich, Ludwig-Maximilians-University of Munich, 80539 Munich, Germany; rudolf.werner@med.uni-muenchen.de
8 Division of Nuclear Medicine and Molecular Imaging, The Russell H Morgan Department of Radiology and Radiological Science, Johns Hopkins University School of Medicine, Baltimore, MD 21205, USA
* Correspondence: thiguchi@me.com; Tel.: +49-931-201-35455

Chemistry

Synthetic procedures and compound characterization

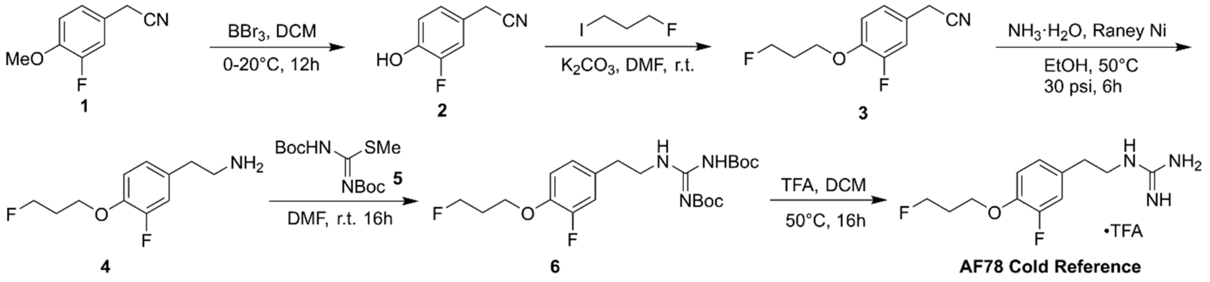

Scheme S1. Modified synthetic scheme of cold reference of [^{18}F]fluproxadine (AF78).

2-(3-Fluoro-4-hydroxyphenyl)acetonitrile (2)

To a solution of 2-(3-fluoro-4-methoxyphenyl)acetonitrile (25 g, 151.36 mmol) in DCM (300 mL), BBr_3 (94.80 g, 378.41 mmol) was added dropwise at 0 °C under N_2. The resulting mixture was stirred at 20 °C for 12 h. The reaction mixture was quenched by pouring into H_2O (300 mL) at 0 °C and extracted with DCM (2 × 100 mL). The combined organic layers were washed with saturated $NaHCO_3$ (300 mL) and brine (300 mL), dried over anhydrous Na_2SO_4, filtered, and concentrated under reduced pressure to give 2-(3-fluoro-4-hydroxy-phenyl)acetonitrile (18.7 g, 82%) as a white solid.

^{1}H NMR ($CDCl_3$, 400 MHz) δ = 7.11–7.06 (m, 1H), 7.03–6.99 (m, 2H), 5.37 (br s, 1H), 3.69 (s, 2H) ppm.

2-(3-Fluoro-4-(3-fluoropropoxy)phenyl)acetonitrile (3)

To a solution of 1-fluoro-3-iodo-propane (29.85 g, 158.80 mmol) in DMF (200 mL), K_2CO_3 (40.24 g, 291.13 mmol) and 2-(3-fluoro-4-hydroxyphenyl)acetonitrile (20 g, 132.33 mmol) were added at 20 °C. The mixture was stirred at 60 °C for 3 h. The reaction mixture was poured into H_2O (300 mL) and then extracted with EtOAc (3 × 100 mL). The combined organic layers were washed with brine (2 × 100 mL), dried over anhydrous Na_2SO_4, filtered, and concentrated under reduced pressure to give 2-(3-fluoro-4-(3-fluoropropoxy)phenyl)acetonitrile (27.9 g, 99%) as a colorless oil.

^{1}H NMR ($CDCl_3$, 400 MHz) δ = 7.10–7.02 (m, 2H), 7.01–6.95 (m, 1H), 4.68 (td, J = 6.4, 47.2 Hz, 2H), 4.18 (t, J = 6.0 Hz, 2H), 3.69 (s, 2H), 2.29-2.14 (m, 2H) ppm.

2-(3-Fluoro-4-(3-fluoropropoxy)phenyl)ethanamine (4)

To a solution of Raney-Ni (2 g, 23.35 mmol) in EtOH (100 mL), 2-(3-fluoro-4-(3-fluoropropoxy)phenyl)acetonitrile (15 g, 71.02 mmol) and $NH_3 \cdot H_2O$ (27.66 g, 213.06 mmol, 30.39 mL, 27%) were added at 20 °C under N_2. The suspension was degassed under vacuum and purged with H_2 3 times. The mixture was stirred under H_2 (30 psi) at 50 °C for 12 h. The mixture was filtered and concentrated under reduced pressure to give 2-(3-fluoro-4-(3-fluoropropoxy)phenyl)ethanamine (13 g, 85%) as a green oil.

^{1}H NMR ($CDCl_3$, 400 MHz) δ = 7.00–6.87 (m, 3H), 4.68 (td, *J* = 6.4, 47.2 Hz, 2H), 4.15 (t, *J* = 6.0 Hz, 2H), 3.68–2.48 (m, 4H), 2.26–2.12 (m, 2H) ppm. LC-MS calculated for $C_{11}H_{15}F_2NO$ *m/z* 216.11 $[M]^+$, found 216.2.

tert-Butyl-_N_-[(_tert_-butoxycarbonylamino)-[2-[3-fluoro-4-(3-fluoropropoxy)phenyl] ethylamino]methylene]carbamate (6)

To a solution of 2-(3-fluoro-4-(3-fluoropropoxy)phenyl)ethanamine (13 g, 60.40 mmol) in DMF (150 mL), *tert*-butyl *N*-[(*tert*-butoxycarbonylamino)-methylsulfanyl-methylene]carbamate (18.42 g, 63.42 mmol) was added at 20 °C. The mixture was stirred at 20 °C for 12 h. The reaction mixture was poured into H_2O (200 mL) and then extracted with EtOAc (3 × 100 mL). The combined organic layers were washed with brine (3 × 100 mL), dried over anhydrous Na_2SO_4, filtered, and concentrated under reduced pressure. The residue was purified by silica gel chromatography (PE : EtOAc = 1:0 to 0:1) to give *tert*-butyl *N*-[(*tert*-butoxycarbonylamino)-[2-[3-fluoro-4-(3-fluoropropoxy)phenyl]ethylamino]methylene]carbamate (26 g, 94%) as a yellow oil.

^{1}H NMR (400 MHz, $CDCl_3$) δ = 11.47 (s, 1H), 8.38 (br s, 1H), 7.00–6.93 (m, 1H), 6.93–6.88 (m, 2H), 4.68 (td, *J* = 6.4, 47.2 Hz, 2H), 4.15 (t, *J* = 6.0 Hz, 2H), 3.70–3.59 (m, 2H), 2.81 (t, *J* = 7.2 Hz, 2H), 2.28–2.12 (m, 2H), 1.51 (s, 9H), 1.48 (s, 9H) ppm.

1-(3-Fluoro-4-(3-fluoropropoxy)phenethyl)guanidine hydrochloride (fluproxadine cold reference)

A mixture of *tert*-butyl *N*-[(*tert*-butoxycarbonylamino)-[2-[3-fluoro-4-(3-fluoropropoxy)phenyl]ethylamino]methylene]carbamate (26 g, 56.83 mmol) in HCl/MeOH (4 M, 200 mL) was stirred at 20 °C for 12 h. Then the mixture was concentrated under reduced pressure. The residue was diluted with DCM (200 mL), and TFA (107.80 g, 945.42 mmol, 70 mL) was added. The mixture was stirred at 50 °C for 12 h. Then the mixture was concentrated under reduced pressure. The residue HCl/EtOAc (4 M, 14.21 mL) was added and stirred at 20 °C for 2 h. Then the mixture was concentrated under reduced pressure to give 1-(3-fluoro-4-(3-fluoropropoxy)phenethyl)guanidine hydrochloride (15 g, 90%) as a pink solid.

^{1}H NMR (DMSO-d_6, 400 MHz) δ = 7.81 (br t, *J* = 5.6 Hz, 1H), 7.68–7.32 (m, 2H), 7.19 (dd, *J* = 2.0, 12.8 Hz, 1H), 7.15–7.08 (m, 1H), 7.06–7.00 (m, 1H), 4.59 (td, *J* = 6.4, 47.2 Hz, 2H), 4.11 (t, *J* = 6.4 Hz, 2H), 3.95–3.66 (m, 1H), 3.38-3.29 (m, 2H), 2.71 (t, *J* = 7.2 Hz, 2H), 2.18–2.01 (m, 2H) ppm. LC-MS calculated for $C_{12}H_{17}F_2N_3O$ *m/z* 258.13 $[M]^+$ found 258.1.

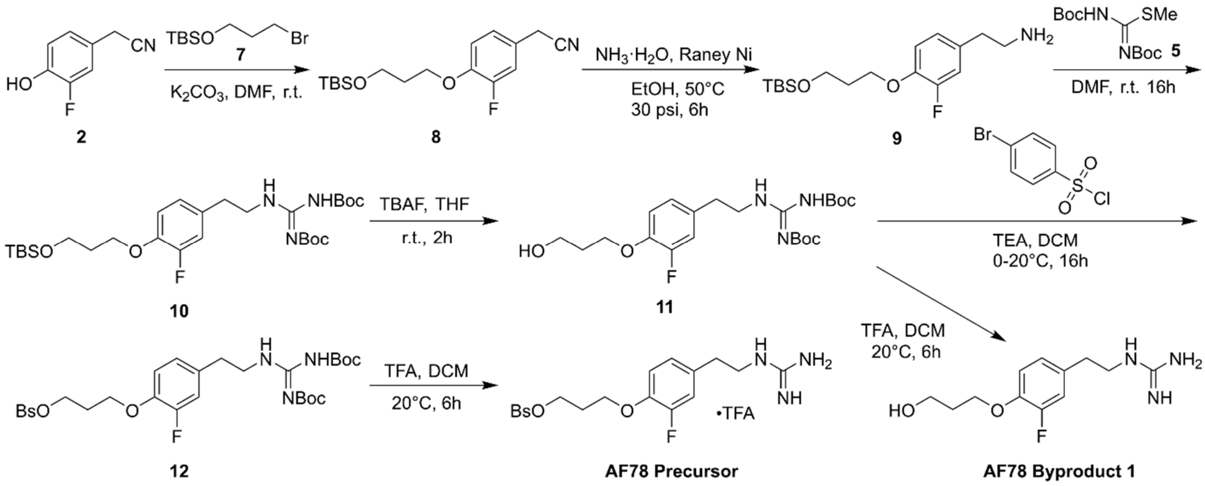

Scheme S2. Synthetic scheme of unprotected precursor of [^{18}F]fluproxadine (AF78) and byproduct 1 decomposed during radiolabeling from its precursor.

2-(4-(3-((*tert*-Butyldimethylsilyl)oxy)propoxy)-3-fluorophenyl)acetonitrile (8)

To a solution of 2-(3-fluoro-4-hydroxy-phenyl)acetonitrile (18.9 g, 125.05 mmol) in DMF (180 mL), K_2CO_3 (43.21 g, 312.63 mmol) and 3-bromopropoxy-tert-butyl-dimethyl-silane (19.00 g, 75.03 mmol) were added at 20 °C. The mixture was stirred at 20 °C for 12 h. The reaction mixture was poured into H_2O (300 mL) and then extracted with MTBE (3 × 100 mL). The combined organic layers were dried over anhydrous Na_2SO_4, filtered, and concentrated under reduced pressure to give a residue. The residue was purified by silica gel chromatography (PE : EtOAc = 100:0 to 70:30) to give 2-(4-(3-((*tert*-butyldimethylsilyl)oxy)propoxy)-3-fluorophenyl)acetonitrile (32 g, 79%) as a colorless oil.

^{1}H NMR ($CDCl_3$, 400 MHz) δ = 7.09–6.94 (m, 3H), 4.17–4.11 (m, 2H), 3.82 (t, J = 6.0 Hz, 2H), 3.69 (s, 2H), 2.04–1.98 (m, 2H), 0.89 (s, 9H), 0.05 (s, 6H) ppm. LC-MS calculated for $C_{17}H_{26}FNO_2Si$ *m/z* 324.17 [M]$^+$ found 324.2.

2-(4-(3-((*tert*-Butyldimethylsilyl)oxy)propoxy)-3-fluorophenyl)ethanamine (9)

Raney nickel (5.20 g, 60.69 mmol) was added in a hydrogenated bottle filled with argon, and ethanol was poured down the wall to moisten it. Then a solution of 2-[4-[3-[*tert*-butyl(dimethyl)silyl]oxypropoxy]-3-fluoro-phenyl]acetonitrile (13 g, 40.19 mmol) in EtOH (100 mL) and $NH_3{\cdot}H_2O$ (30 mL) was added under Argon gas. The suspension was degassed under vacuum and purged with H_2 several times. The mixture was stirred under H_2(30 psi) at 50°C for 16 h. The mixture was filtered and the filtrate was concentrated under reduced pressure to give 2-(4-(3-((*tert*-butyldimethylsilyl)oxy)propoxy)-3-fluorophenyl)ethanamine (26 g, 98%) as a light blue oil.

^{1}H NMR (DMSO-d_6, 400 MHz) δ = 7.08–7.00 (m, 2H), 6.92 (br d, J = 8.0 Hz, 1H), 4.06 (t, J = 6.0 Hz, 2H), 3.74 (t, J = 6.0 Hz, 2H), 2.75–2.53 (m, 4H), 1.88 (quin, J = 6.0 Hz, 2H), 0.84 (s, 9H), 0.01 (s, 6H) ppm. LC-MS calculated for $C_{17}H_{30}FNO_2Si$ *m/z* 328.20 [M]$^+$ found 328.2.

***tert*-Butyl *N*-[(*tert*-butoxycarbonylamino)-[2-[4-[3-[*tert*-butyl(dimethyl)silyl]oxypropoxy]-3-fluoro-phenyl]ethylamino]methylene]carbamate (10)**

To a solution of 2-[4-[3-[*tert*-butyl(dimethyl)silyl]oxypropoxy]-3-fluoro-phenyl]ethanamine (29 g, 88.55 mmol) in DMF (300 mL), *tert*-butyl *N*-[(*tert*-butoxycarbonylamino)-methylsulfanyl-methylene]carbamate (25.71 g, 88.55 mmol) was added at 20°C. The mixture was stirred at 20°C for 16 h. The reaction mixture was poured into H_2O (1000 mL) and then extracted with EtOAc (3 × 300 mL). The combined organic layers were dried over anhydrous Na_2SO_4, filtered, and concentrated under reduced pressure to give a residue. The residue was purified by silica gel chromatography (PE : EtOAc = 100:0 to 90:10) to give *tert*-butyl *N*-[(*tert*-butoxycarbonylamino)-[2-[4-[3-[*tert*-butyl(dimethyl)silyl]oxypropoxy]-3-fluoro-phenyl]ethylamino]methylene]carbamate (40 g, 79%) as a white solid.

^{1}H NMR ($CDCl_3$, 400 MHz) δ = 11.47 (s, 1H), 8.38 (br s, 1H), 7.08–6.74 (m, 3H), 4.12 (t, *J* = 6.4 Hz, 2H), 3.82 (t, *J* = 6.0 Hz, 2H), 3.71–3.60 (m, 2H), 2.80 (t, *J* = 7.2 Hz, 2H), 2.04–1.98 (m, 2H), 1.51 (s, 9H), 1.48 (s, 9H), 0.89 (s, 9H), 0.05 (s, 6H) ppm. LC-MS calculated for $C_{28}H_{48}FN_3O_6Si$ *m/z* 570.33 $[M]^+$ found 570.3.

***tert*-Butyl *N*-[(*tert*-butoxycarbonylamino)-[2-[3-fluoro-4-(3-hydroxypropoxy)phenyl] ethylamino]methylene]carbamate (11)**

To a mixture of *tert*-butyl *N*-[(*tert*-butoxycarbonylamino)-[2-[4-[3-[*tert*-butyl(dimethyl) silyl]oxypropoxy]-3-fluoro-phenyl]ethylamino]methylene]carbamate (40 g, 70.21 mmol) in THF (400 mL), TBAF (1 M, 140.4 mL) (1 M in THF) was added at 0°C under N_2. The mixture was stirred at 20 °C for 4 h. The reaction mixture was poured into H_2O (1000 mL) and then extracted with EtOAc (3 × 300 mL). The combined organic layers were dried over anhydrous Na_2SO_4, filtered, and concentrated under reduced pressure to give a residue. The residue was purified by silica gel chromatography (PE : EtOAc = 100:0 to 0:100) to give *tert*-butyl *N*-[(*tert*-butoxycarbonylamino)-[2-[3-fluoro-4-(3-hydroxypropoxy)phenyl]ethylamino] methylene]carbamate (28.5 g) as a colorless oil.

^{1}H NMR ($CDCl_3$, 400 MHz) δ = 11.47 (s, 1H), 8.36 (br s, 1H), 7.00–6.87 (m, 3H), 4.19 (t, *J* = 6.0 Hz, 2H), 3.89 (q, *J* = 5.6 Hz, 2H), 3.69–3.59 (m, 2H), 2.81 (t, *J* = 7.2 Hz, 2H), 2.11–2.05 (m, 2H), 1.86 (t, *J* = 5.2 Hz, 1H), 1.51 (s, 9H), 1.48 (s, 9H) ppm. LC-MS calculated for $C_{22}H_{34}FN_3O_6$ *m/z* 456.24 $[M]^+$ found 456.2.

3-(4-(2-(2,3-Bis(*tert*-butoxycarbonyl)guanidino)ethyl)-2-fluorophenoxy)propyl 4-bromobenzenesulfonate (12)

To a mixture of *tert*-butyl *N*-[(*tert*-butoxycarbonylamino)-[2-[3-fluoro-4-(3-hydroxypropoxy)phenyl]ethylamino]methylene]carbamate (28 g, 61.25 mmol) in DCM (280 mL), TEA (12.4 g, 122.5 mmol) and 4-bromobenzenesulfonyl chloride (18.79 g, 73.50 mmol) were added at 0 °C under N_2. The mixture was stirred at 2–4 °C for 16 h. The reaction mixture was poured into H_2O (800 mL) and then extracted with EtOAc (3 × 200 mL). The combined organic layers were dried over anhydrous Na_2SO_4, filtered, and concentrated under reduced pressure to give a residue. The residue was purified by silica gel chromatography (PE : EtOAc = 100:0 to 40:60) and then the residue was triturated with PE : MTBE (90 mL,

3:1), filtered, and the filter cake was dried under reduced pressure to give 3-(4-(2-(2,3-bis(*tert*-butoxycarbonyl)guanidino)ethyl)-2-fluorophenoxy)propyl 4-bromobenzenesulfonate (33 g, 80%) as a white solid.

^{1}H NMR (400 MHz, $CDCl_3$) δ = 11.48 (s, 1H), 8.38 (br s, 1H), 7.76–7.70 (m, 2H), 7.62–7.54 (m, 2H), 6.95 (dd, *J* = 2.0, 12.0 Hz, 1H), 6.89 (br d, *J* = 8.4 Hz, 1H), 6.80–6.70 (m, 1H), 4.31 (t, *J* = 5.6 Hz, 2H), 3.98 (t, *J* = 6.0 Hz, 2H), 3.72–3.59 (m, 2H), 2.82 (t, *J* = 7.2 Hz, 2H), 2.18–2.05 (m, *J* = 6.0 Hz, 2H), 1.51 (s, 9H), 1.48 (s, 9H) ppm. LC-MS calculated for $C_{28}H_{37}BrFN_3O_8S$ *m/z* 674.15, 676.15 $[M]^+$ found 674.1, 676.1.

3-(2-Fluoro-4-(2-guanidinoethyl)phenoxy)propyl 4-bromobenzenesulfonate (fluproxadine precursor)

To a solution of 3-[4-[2-[[*N*,*N*'-bis(*tert*-butoxycarbonyl)carbamimidoyl]amino]ethyl]-2-fluoro-phenoxy]propyl 4-bromobenzenesulfonate (23 g, 34.10 mmol) in DCM (230 mL), TFA (45 mL) was added at 20 °C under N_2. The mixture was stirred at 20 °C for 16 h. The reaction was concentrated under reduced pressure to remove DCM and TFA to give a residue. The residue was triturated with MTBE (50 mL) and filtered. The filter cake was dried under reduced pressure to give 3-(2-fluoro-4-(2-guanidinoethyl)phenoxy)propyl 4-bromobenzenesulfonate (18 g, TFA salt) as a white solid.

^{1}H NMR (400 MHz, CD_3OD) δ = 7.76 (d, *J* = 7.2 Hz, 2H), 7.67 (d, *J* = 7.2 Hz, 2H), 7.06–6.87 (m, 3H), 4.29 (t, *J* = 5.6 Hz, 2H), 3.98 (t, *J* = 5.6 Hz, 2H), 3.43 (t, *J* = 7.2 Hz, 2H), 2.83 (t, *J* = 7.2 Hz, 2H), 2.17–2.05 (m, 2H) ppm. LC-MS calculated for $C_{18}H_{21}BrFN_3O_4S$ *m/z* 474.04, 476.04 $[M]^+$ found 473.9, 475.9.

1-(3-Fluoro-4-(3-hydroxypropoxy)phenethyl)guanidine (fluproxadine byproduct 1)

A solution of *tert*-butyl *N*-[(*tert*-butoxycarbonylamino)-[2-[3-fluoro-4-(3-hydroxypropoxy)phenyl]ethylamino]methylene]carbamate (100 mg, 0.22 mmol) in DCM (2 mL) and TFA (0.2 mL) was stirred at 20 °C for 2 h. The reaction mixture was concentrated under reduced pressure. The residue was purified by prep-HPLC (column Phenomenex luna C18 (75 × 30 mm × 3 µm); mobile phase: A: 0.1% TFA in water; B: MeCN; B% in A: 1%–30%, 8 min) to give 1-(3-fluoro-4-(3-hydroxypropoxy)phenethyl)guanidine (24.31 mg, 43 %, 100% purity).

^{1}H-NMR (400 MHz, CD_3OD) δ = 7.08–6.94 (m, 3H), 4.13 (t, *J* = 6.4 Hz, 2H), 3.75 (t, *J* = 6.4 Hz, 2H), 3.41 (t, *J* = 7.2 Hz, 2H), 2.81 (t, *J* = 7.2 Hz, 2H), 1.99 (t, *J* = 6.0 Hz, 2H). LC-MS calculated for $C_{12}H_{19}FN_3O_2$ *m/z* = 256.15 $[M+H]^+$ found 256.1.

Radiochemistry

Table S1. Summary of screening of radiolabeling conditions.

**Base/elution from QMA**	**Solvent**	**Amount / concentration of precursor**	**Temperature / time**	**RCY (d.c.)**
K_2CO_3 (**1.1** eq.), K_{222} (2.2 eq.)	300 µL 4:1 tBuOH/MeCN	2.4 mg (8 mg/mL)	110°C, 15 min	16%
K_2CO_3 (**1.5** eq.), K_{222} (3 eq.)	300 µL 4:1 tBuOH/MeCN	2.4 mg (8 mg/mL)	110°C, 15 min	33%
K_2CO_3 (**2.0** eq.), K_{222} (4 eq.)	300 µL 4:1 tBuOH/MeCN	2.4 mg (8 mg/mL)	110°C, 15 min	33%
K_2CO_3 (**2.5** eq.), K_{222} (5 eq.)	300 µL 4:1 tBuOH/MeCN	2.4 mg (8 mg/mL)	110°C, 15 min	27%
K_2CO_3 (1.5 eq.), K_{222} (3 eq.)	300 µL **4:1** tBuOH/MeCN	2.4 mg (8 mg/mL)	110°C, 15 min	33%
K_2CO_3 (1.5 eq.), K_{222} (3 eq.)	300 µL **3:1** tBuOH/MeCN	2.4 mg (8 mg/mL)	110°C, 15 min	31%
K_2CO_3 (1.5 eq.), K_{222} (3 eq.)	300 µL **2:1** tBuOH/MeCN	2.4 mg (8 mg/mL)	110°C, 15 min	21%
K_2CO_3 (1.5 eq.), K_{222} (3 eq.)	300 µL **1:1** tBuOH/MeCN	2.4 mg (8 mg/mL)	110°C, 15 min	29%
K_2CO_3 (1.5 eq.), K_{222} (3 eq.)	300 µL **1:2** tBuOH/MeCN	2.4 mg (8 mg/mL)	110°C, 15 min	16%
K_2CO_3 (1.5 eq.), K_{222} (3 eq.)	300 µL 4:1 tBuOH/MeCN	**1.8** mg (6 mg/mL)	110°C, 15 min	22%
K_2CO_3 (1.5 eq.), K_{222} (3 eq.)	300 µL 4:1 tBuOH/MeCN	**2.4** mg (8 mg/mL)	110°C, 15 min	33%
K_2CO_3 (1.5 eq.), K_{222} (3 eq.)	300 µL 4:1 tBuOH/MeCN	**3.0** mg (10 mg/mL)	110°C, 15 min	31%

K_2CO_3 (1.5 eq.), K_{222} (3 eq.)	300 μL 4:1 tBuOH/MeCN	2.4 mg (8 mg/mL)	**90**°C, 15 min	28%
K_2CO_3 (1.5 eq.), K_{222} (3 eq.)	300 μL 4:1 tBuOH/MeCN	2.4 mg (8 mg/mL)	**100**°C, 15 min	32%
K_2CO_3 (1.5 eq.), K_{222} (3 eq.)	300 μL 4:1 tBuOH/MeCN	2.4 mg (8 mg/mL)	**110**°C, 15 min	33%
K_2CO_3 (1.5 eq.), K_{222} (3 eq.)	300 μL 4:1 tBuOH/MeCN	2.4 mg (8 mg/mL)	**120**°C, 15 min	28%
K_2CO_3 (1.5 eq.), K_{222} (3 eq.)	300 μL 4:1 tBuOH/MeCN	2.4 mg (8 mg/mL)	110°C, **10** min	30%
K_2CO_3 (1.5 eq.), K_{222} (3 eq.)	300 μL 4:1 tBuOH/MeCN	2.4 mg (8 mg/mL)	110°C, **15** min	33%
K_2CO_3 (1.5 eq.), K_{222} (3 eq.)	300 μL 4:1 tBuOH/MeCN	2.4 mg (8 mg/mL)	110°C, **20** min	32%

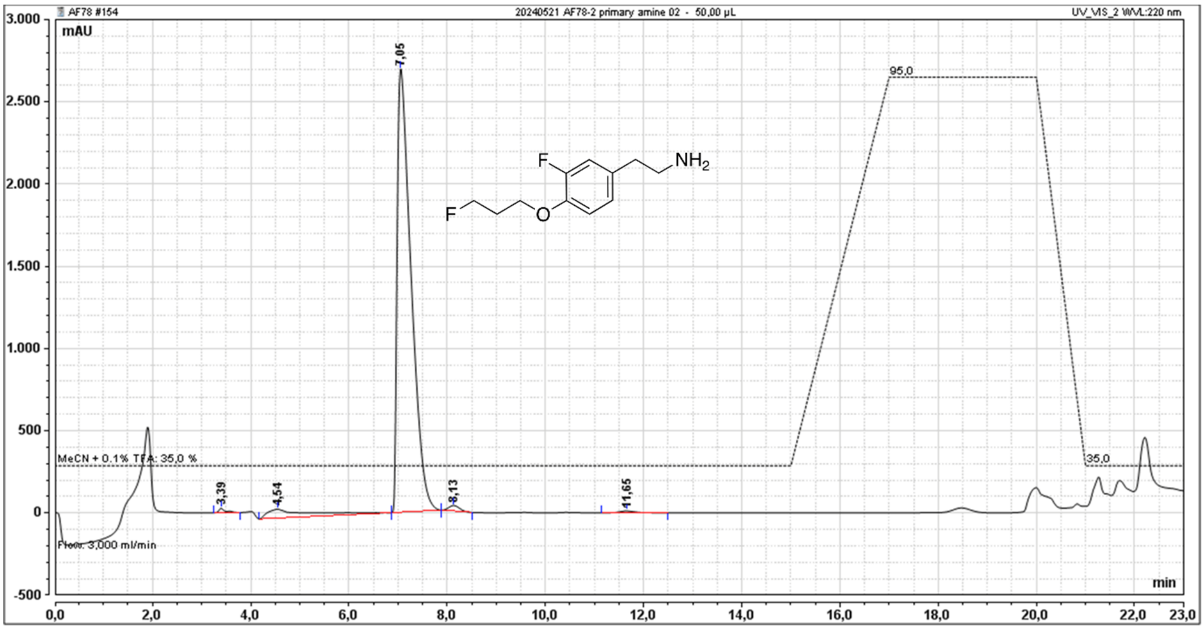

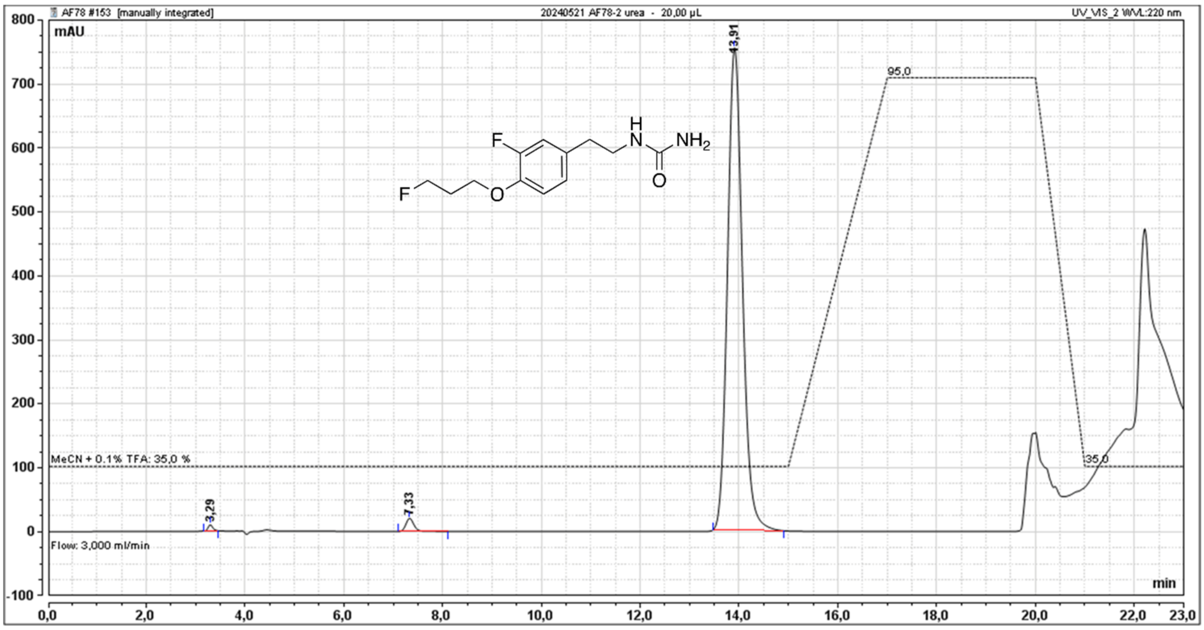

Figure S1. HPLC chromatographies of potential byproducts, ethylamine and urea, decomposed from [^{18}F]fluproxadine under basic conditions during the radiolabeling process. Phenomenex Synergi Hydro-RP 5 µm 10ID × 250 mm, 35% isocratic MeCN/H_2O with 0.1%TFA.

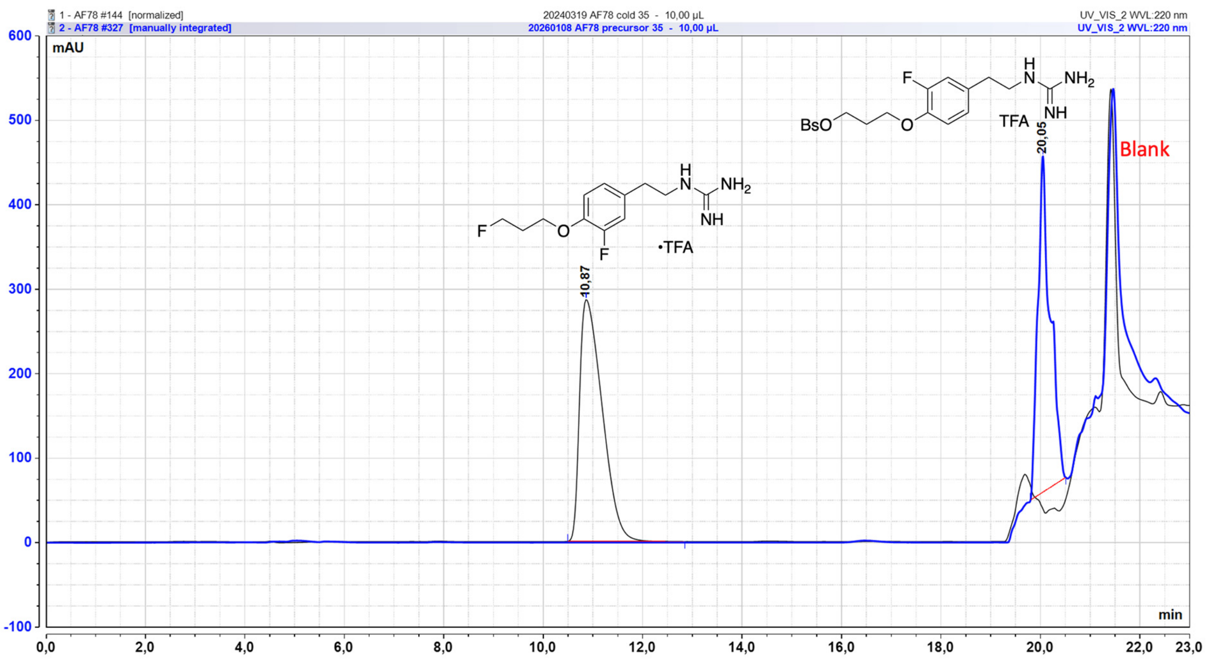

Figure S2. HPLC chromatographies of cold standard of [^{18}F]fluproxadine and corresponding precursor at purification conditions via HPLC. Phenomenex Synergi Hydro-RP 5 µm 10ID × 250 mm, 35% isocratic MeCN/H_2O with 0.1%TFA. [^{18}F]fluproxadine RT 10.87 min, precursor RT 20.05 min.

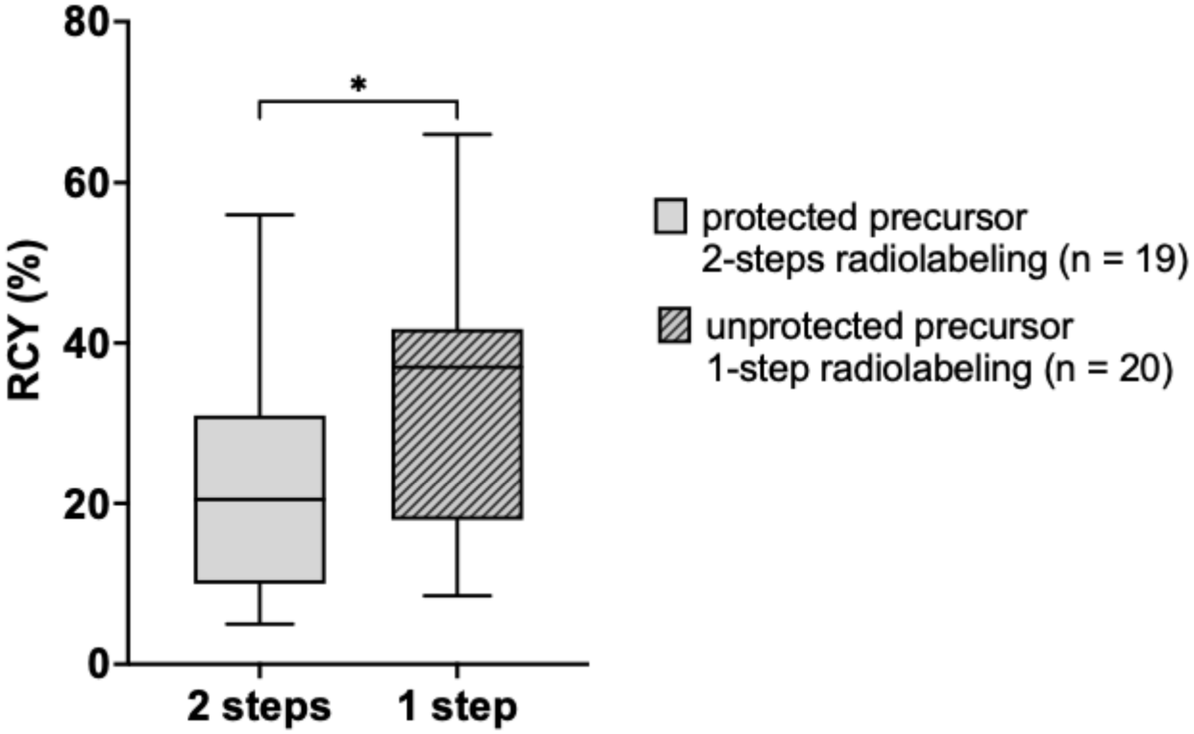

Figure S3. Radiochemical yields (RCYs) for [^{18}F]fluproxadine labeling varied significantly depending on the precursor and labeling protocol used. The 1-step radiolabeling protocol utilizing an unprotected precursor yielded significantly higher RCYs (32.6 ± 15.3%, n = 20) compared to the 2-step radiolabeling protocol employing a fully protected guanidine precursor (22.6 ± 14.1%, n = 19). *$P < 0.05$.
